# Desnutrição e Caquexia: A Importância da Avaliação na Cardiomiopatia Chagásica

**DOI:** 10.36660/abc.20210919

**Published:** 2022-01-01

**Affiliations:** 1 Universidade Federal de Goiás Hospital das Clínicas Goiânia GO Brasil Hospital das Clínicas da Universidade Federal de Goiás, Goiânia, GO - Brasil

**Keywords:** Desnutrição, Caquexia, Insuficiência Cardíaca/complicações, Cardiomiopatia Chagásica, Morbimortalidade, Epidemiologia, Hospitalização, Perda de Peso, Prognóstico

A cardiomiopatia chagásica, descrita por Carlos Chagas há mais de 100 anos, é ainda causa de significativa morbimortalidade e desempenha papel negativo socioeconômico em países da América Latina, entre eles o Brasil.^1^

A desnutrição e a caquexia em pacientes com insuficiência cardíaca (IC), em especial de etiologia chagásica, são multifatoriais. A redução na ingestão alimentar pode ser secundária à oferta diminuída de alimentos em uma população de pacientes usualmente em situação financeira desfavorável ou à anorexia, comum nessa síndrome de IC.^2^ Vários fatores podem estar envolvidos na anorexia e perda de peso, como dietas pouco saborosas (principalmente pelo baixo teor de sódio) e o estado congestivo visceral, principalmente hepático e intestinal. A hepatomegalia, frequentemente volumosa, leva a um desconforto gástrico pelo aumento do lobo esquerdo do fígado e compressão do estômago. A dor epigástrica no hipocôndrio direito deve-se à distensão da cápsula hepática. O edema da mucosa intestinal causa má absorção intestinal de proteínas e gorduras, e piora da nutrição.^3^ Alterações neuroendócrinas e imunológicas também estão envolvidos no desenvolvimento da caquexia no paciente com IC.^4^ Pacientes com caquexia tem concentrações plasmáticas elevadas de fator de necrose tumoral alfa (TNF-alfa) e outras citocinas infamatórias, particularmente a interleucina (IL) −6 e IL-1.^4^ Isso decorre do fenômeno de translocação bacteriana, no qual as anormalidades do trato intestinal estão envolvidas no desenvolvimento da caquexia e da ativação inflamatória sistêmica. Os efeitos dessas citocinas incluem proteólise e perda de massa muscular esquelética, com agravamento da caquexia.^4^

A ativação neuro-humoral decorrente do baixo débito sistêmico na IC na cardiomiopatia chagásica, leva a uma elevação plasmática de noradrenalina, angiotensina II e aldosterona. O adequado tratamento com medicações que bloqueiam esses neuro-hormônios diminui as chances do desenvolvimento de caquexia cardíaca.^2,5-8^

Neste estudo bem elaborado, Tavares et al.^6^ correlacionaram a ocorrência de desnutrição e caquexia na IC descompensada de etiologia chagásica com desfechos hospitalares. É sabido que pacientes em IC de etiologia chagásica tem pior prognóstico que o de outras etiologias;^7^ entretanto, o quanto as características nutricionais impactam no prognóstico desses pacientes é ainda pouco estudado. Assim, a hipótese dos autores,^6^ de que os distúrbios nutricionais sejam comuns nos pacientes com IC descompensada e tenham impacto no prognóstico, o qual seria diferente de acordo com a etiologia da IC.

Foi realizado um estudo de série de casos consecutivos, com pacientes hospitalizados com IC descompensada. Os pacientes foram avaliados quanto à ocorrência de caquexia e baixa massa muscular e força por Avaliação Nutricional Subjetiva Global (ASG) e medidas antropométricas e laboratoriais, e quanto à ocorrência de morte e transplante cardíaco de urgência durante a internação. Foram analisados 131 pacientes consecutivos, sendo que 42 (32,1%) tinham doença de Chagas. Esses apresentavam Índice de Massa Corporal (IMC) menor que pacientes sem doença de Chagas (22,4kg/m^2^ vs. 23,6kg/m^2^ p=0,003) e maior frequência de desnutrição (76,2% vs 55,1 p=0,015) além de maior ocorrência de morte e transplante cardíaco (83,3% vs 41,6% p<0,001).

A partir dos resultados, a autora concluiu que pacientes com doença de Chagas internados com IC descompensada costumam apresentar problemas nutricionais, principalmente desnutrição, os quais estão associados à maior ocorrência de morte e transplante cardíaco durante a internação.^8^

Os dados do estudo mostraram que os pacientes com cardiomiopatia chagásica apresentavam uma doença mais grave, com níveis séricos mais elevados de peptídeo natriurético cerebral e piores desfechos hospitalares. Também apresentavam pior **status** nutricional, representado por baixo peso corporal e IMC, perda de massa muscular e maior frequência de desnutrição na ASG.

Devemos lembrar que os pacientes com IC de etiologia chagásica tem frequentemente predomínio de falência cardíaca direita, com hepatomegalia, edema de alças intestinais e, consequentemente, má-absorção proteica e maior atividade inflamatória intestinal.^7,9^ Portanto, estes fatores: maior gravidade do distúrbio hemodinâmico, edema de alça intestinal e atividade inflamatória exacerbada têm impacto maior na ocorrência de desnutrição, reforçando a necessidade de uma avaliação e suporte nutricional mais adequado, principalmente visando melhores resultados clínicos no tratamento.

Este ensaio clínico nos traz informações importantes, mas não é um estudo randomizado de intervenção terapêutica. O estudo ainda reforça o impacto da desnutrição e caquexia na cardiomiopatia chagásica, independente da presença de megaesôfago, que foi critério de exclusão neste estudo.
