# Supplementary material for: Desnutrição e Caquexia: A Importância da Avaliação na Cardiomiopatia Chagásica
Source: Arq Bras Cardiol. 2022 Jan 1;118(1):12–3. [Article in Portuguese] doi: 10.36660/abc.20210919 (PMC8959061; doi:10.36660/abc.20210919)
Supplement: Translation

# Translation: The Importance of Assessing Malnutrition and Cachexia in Chagas Cardiomyopathy

**Affiliations:** 1 Goiânia GO Brazil Hospital das Clínicas da Universidade Federal de Goiás, Goiânia, GO - Brazil

**Keywords:** Malnutrition, Cachexia, Heart Failure/complications, Chagas Cardiomyopathy, Morbimortality, Epidemiology, Hospitalization, Weight Loss, Prognosis

Chagas cardiomyopathy, described by Carlos Chagas more than 100 years ago, is still a cause of significant morbidity and mortality, and has a negative effect on socioeconomic status of Latin America countries, including Brazil.^1^

Malnutrition and cachexia in heart failure (HF), especially in Chagas disease patients, has a multifactorial etiology. Decreased food intake may be caused either by a reduced supply of foods to these usually underprivileged patients or by anorexia, which is common in this syndrome.^2^ Many factors may be involved in anorexia and weight loss, including tasteless food (mainly due to the low sodium diet) and due to visceral (mainly hepatic and intestinal) congestion. Hepatomegaly, usually dense, causes gastric discomfort due to left lobe enlargement and compression of the stomach. Pain in the right hypochondrium is caused by stretching of the liver capsule. Intestinal mucosal edema causes protein and fat malabsorption, ultimately affecting nutrition.^3^ Neuroendocrine and immunological changes are also involved in the development of cachexia in HF patients.^4^ Patients with cachexia have increased plasma levels of tumor necrosis factor-alpha (TNF-α) and other inflammatory cytokines, particularly interleukin (IL)-6 and IL-1.^4^ This results from bacterial translocation, in which abnormalities of the gastrointestinal tract are involved in the development of cachexia and systemic inflammation. Effects of these cytokines include proteolysis and loss of skeletal muscle mass, with aggravation of cachexia.^4^

Neurohumoral activation resulting from diminished systemic output in Chagas cardiomyopathy-related HF causes an increase in plasma noradrenaline, angiotensin II, and aldosterone. An adequate treatment with drugs that block these neurohormones reduces the risk of cardiac cachexia.^2,5–8^

In this well-designed study, Tavares LCA.^6^ correlated malnutrition and cachexia in patients with Chagas disease and decompensated HF with hospital outcomes. It is known that patients with HF caused by Chagas disease have worse prognosis than other etiologies;^7^ however, the impact of nutritional factors on the prognosis of these patients is still poorly understood. Therefore, the hypothesis of the authors^6^ is that nutritional disorders are common in patients with decompensated HF and have in impact on prognosis, which would be different according to the etiology of HF.

The study was conducted on a consecutive series of patients hospitalized for decompensated HF. Patients were assessed for nutritional status by subjective global assessment (SGA), and anthropometric and laboratory measurements, and for urgent heart transplantation and death during hospitalization. A total of 131 consecutive patients were analyzed, 42 (32.1%) of them had Chagas disease. Patients with Chagas disease had lower body mass index (BMI) (22.4kg/m² vs. 23.6kg/m² p=0.003) and higher frequency of malnutrition (76.2% vs. 55.1 p=0.015), death and cardiac transplantation (83.3% vs. 41.6% p<0.001) compared with patients without the disease.

Based on the results, the author concluded that Chagas disease patients admitted with decompensated HF commonly have nutritional problems, especially malnutrition, that are associated with higher frequency of death and cardiac transplantation during hospitalization.^8^

The study showed that the patients with Chagas cardiomyopathy had more severe illness, with increased brain natriuretic peptide levels and worse hospital outcomes. These patients also had worse nutritional status, namely low body weight and BMI, loss of muscle mass and more malnutrition according to the SGA.

We must remember that Chagas disease patients with HF mostly present with right heart failure, with hepatomegaly, edema of intestinal loops and, consequently, protein malabsorption and increased intestinal inflammatory activity.^7,9^ Thus, the high severity of hemodynamic dysfunction, edema of intestinal loop, and exacerbated inflammation have greater impact on malnutrition, reinforcing the need for adequate nutritional assessment and support, especially focusing on better treatment outcomes.

Although this clinical trial provides important information, this is not a randomized intervention study. The study also reinforces the impact of malnutrition and cachexia on Chagas cardiomyopathy, regardless of the presence of megaesophagus, which was an exclusion criterion of the study.
